# A pesticide residue detection model for food based on NIR and SERS

**DOI:** 10.1371/journal.pone.0320456

**Published:** 2025-04-08

**Authors:** Fuchao Yan, Rui Zhang, Shuqi Wang, Ning Zhang, Xueyao Zhang

**Affiliations:** 1 Harbin Children Pharmaceutical Factory, Harbin, Heilongjiang, China; 2 Heilongjiang Institute of Quality Supervision and Testing, Harbin, Heilongjiang, China; 3 Heilongjiang Centre Testing International, Harbin, Heilongjiang, China; 4 Suzhou Standard Testing Group, Suzhou, Jiangsu, China; Purdue University, UNITED STATES OF AMERICA

## Abstract

This paper presents a multivariate calibration model based on Near Infrared Spectroscopy (NIR) and Surface Enhanced Raman Spectroscopy (SERS) techniques, aiming to achieve efficient and accurate detection of pesticide residues in food by integrating the spectral information from both techniques. The study utilizes the Hilbert-Schmidt Independence Criterion-based Variable Space Iterative Optimization algorithm (HSIC-VSIO) for feature variable selection, and combines it with Partial Least Squares Regression (PLSR) to build a spectral fusion quantitative model. Experimental results show that the calibration set Root Mean Square Error (RMSE1) of the NIR and SERS feature-layer fusion model is 0.160, the prediction set RMSE (RMSE2) is 0.185, the prediction set coefficient of determination (R²) is 0.988, and the Relative Percent Deviation (RPD) is 8.290. Compared to single spectral techniques, the NIR and SERS spectral feature-layer fusion method demonstrates significant superiority in detecting pesticide residues in complex matrix samples. The findings further validate the high sensitivity of SERS technology in detecting low concentrations of pesticides and show that the feature-layer fusion method effectively suppresses matrix interference, enhancing the model’s generalization ability. This study provides a reliable tool for the rapid and accurate detection of pesticide residues in food and offers new insights into the application of spectral analysis technologies in the food safety field.

## 1. Introduction

Pesticides play an important role in agricultural production by reducing the damage caused by pests, weeds, and diseases, significantly increasing crop yields, and having vital practical significance in ensuring food security and promoting agricultural economic growth [1]. However, the issue of pesticide residue has become increasingly severe. With the continuous growth of market demand, some growers, in pursuit of higher yields, overuse pesticides. The excessive use of pesticides results in the accumulation of undecomposed pesticides and toxic metabolites in organisms and the environment, which are transferred through the food chain into animals and may ultimately end up in humans. Once pesticide residues accumulate to a certain level in the human body, they pose a direct threat to human health [2]. Meanwhile, pesticide residues in the environment enter lakes, oceans through precipitation, and irrigate farmland, circulating within the agricultural ecosystem, causing long-term food safety issues [3]. Existing studies have confirmed that pesticides are associated with various health problems due to their toxicity. Long-term accumulation in the human body can lead to poisoning, irreversible organ damage, chronic diseases, and even cancer. Short-term ingestion of high doses of pesticides can be fatal [3–5]. Therefore, there is an urgent need to explore precise and efficient detection methods to reduce the risk of pesticide residues in food and mitigate harm to human health.

Fast and accurate pesticide residue detection has become a key research focus. In recent years, spectroscopy has been widely applied in pesticide residue detection due to its simple operation, convenient and non-destructive detection, and the fact that it does not require labeling [6,7]. The principle of spectroscopy is that the light source emits radiation signals, causing transitions in the electronic energy levels of the substrate. These transitions are used to identify chemical components through radiation and conduct qualitative or quantitative analysis based on the relationship between signal intensity and corresponding concentration [8]. Among them, Near Infrared Spectroscopy (NIR) and Surface Enhanced Raman Spectroscopy (SERS) technologies have rapidly developed in the field of food safety due to their convenience, low detection cost, non-reagent contamination, and non-destructive nature to the sample [9,10]. However, the application of these two technologies in pesticide residue detection still requires further research, and most related studies currently employ single techniques. From a theoretical perspective, NIR detects vibrations caused by changes in the electric dipole moment, while SERS detects vibrations caused by molecular polarization. These two types of spectral information are complementary in expressing molecular information [11]. Therefore, integrating these two spectral data to achieve information complementarity is a necessary approach to improve detection accuracy.

In light of this, this study focuses on the detection of pesticide residues in food. During the research process, Near Infrared Spectroscopy (NIR) and Surface Enhanced Raman Spectroscopy (SERS) spectra of food samples were separately collected. The Hilbert-Schmidt Independence Criterion-based Variable Space Iterative Optimization algorithm (HSIC-VSIO) was used to select feature variables from the NIR and SERS spectra. These feature variables were then fused, and based on this, a multivariate calibration model was constructed to achieve fast and highly accurate quantitative detection of pesticide residues in food. The study integrates NIR and SERS technologies to develop an efficient, sensitive, and low-cost pesticide residue detection method. This method significantly enhances the speed and accuracy of food safety detection, enabling regulatory agencies and production enterprises to quickly identify food with excessive pesticide residues and take timely measures to prevent harmful food from entering the market, thus safeguarding public health. The research also introduces the Hilbert-Schmidt Independence Criterion-based Variable Space Iterative Optimization algorithm (HSIC-VSIO), an advanced feature selection method that overcomes the limitations of single spectral technologies by selecting and fusing NIR and SERS spectral feature variables, improving the performance of the detection model. By constructing this multivariate calibration model, the study not only enriches the theoretical application of spectral analysis technologies but also provides more reliable tools for practical detection. This innovative approach expands the application prospects of spectral analysis technology in food safety detection, advancing the development of this field. Overall, this study opens new directions for pesticide residue detection and is of significant importance for ensuring food safety and public health.

## 2. Literature review

### 2.1. Common detection methods for pesticide residues

The purpose of pesticide residue detection is to analyze the pesticide residues in samples through field or extraction methods to obtain qualitative or quantitative data, which are then compared with national standards to evaluate the safety of the samples. Currently, a variety of pesticide residue detection technologies have been developed to achieve trace detection. Among them, the most commonly used methods are chromatography, especially gas chromatography (GC) and high-performance liquid chromatography (HPLC) [12].

The principle of gas chromatography is to separate components based on differences in their adsorption or dissolution abilities in the stationary phase, and display the chromatographic peaks on a recorder after a certain period. Gas chromatography (GC) is often used to detect pesticide residues, particularly in complex matrix samples [13]. Meng et al. (2021) proposed a rapid screening method by combining the multi-plug filtration purification method with gas chromatography-electrostatic field orbitrap high-resolution mass spectrometry (GC-Orbitrap/MS) technology to detect 350 pesticide residues in vegetables and fruit juices. This method not only accelerates the detection speed but also significantly reduces the detection costs [14]. Ferracane et al. (2021) explored the use of flow modulation comprehensive two-dimensional gas chromatography-triple quadrupole mass spectrometry (GC×GC-QqQ-MS) to detect pesticide residues in four vegetable products, including 88 pesticides [15]. Xu et al. (2022) developed a multi-residue analysis method based on gas chromatography-tandem mass spectrometry (GC-MS/MS) for the detection of 77 pesticides in ginseng [16].

High-performance liquid chromatography (HPLC) uses an efficient fixed-phase column chromatography method for the quantitative analysis of target components and is not limited by sample volatility or thermal stability, making it more widely applicable [17]. Tsagkaris et al. (2022) designed and tested an ultra-high-performance liquid chromatography-triple quadrupole mass spectrometry (UHPLC-QqQ-MS) method for the detection of 97 organophosphates and carbamate pesticides in cereals [18]. Wu et al. (2022) developed a method based on integrated automatic extraction and purification technology combined with ultra-high-performance liquid chromatography-quadrupole orbitrap high-resolution mass spectrometry (UHPLC-Q-Orbitrap-MS) for detecting 206 pesticide residues in forage [19]. Oprita et al. (2022) summarized the validation studies of multi-residue methods for pesticide residue detection in fruits and vegetables and evaluated the monitoring control program in Romania using liquid chromatography-mass spectrometry (LC-MS/MS) technology [20].

In quantitative detection, chromatographic methods often have higher sensitivity and can yield accurate results. However, these detection technologies also have drawbacks. Their operation is relatively complex and requires operators to have sufficient professional expertise. In addition, organic reagents used during chromatographic detection may cause environmental pollution. Furthermore, the entire analysis process takes a long time, and the detection costs are high. Therefore, this technology is often preferred for laboratory testing projects, but it is difficult to meet the demand for rapid large-scale detection of agricultural products in market regulation.

### 2.2. Application of Near-Infrared Spectroscopy (NIRS) in food safety

Near-Infrared Spectroscopy (NIRS) is an analytical technique that utilizes electromagnetic waves in the near-infrared region (700–2500 nm) to interact with materials, enabling rapid and non-destructive detection of the molecular composition and structure of samples [21]. In recent years, the application of NIRS in pesticide residue detection has become increasingly widespread, demonstrating its significant potential in the fields of agriculture and food safety.

The outstanding performance of NIRS in pesticide residue detection is attributed to its several notable advantages. NIRS technology allows for rapid and non-destructive testing, making it particularly suitable for situations where maintaining the integrity of the sample is necessary [22]. Moreover, when combined with modern data processing and machine learning algorithms, or with new sensors, NIRS can significantly enhance the accuracy and sensitivity of the detection [23].

In specific applications, researchers have demonstrated the effectiveness of NIRS technology in various agricultural products. Zhang et al. (2023) found that by combining visible/near-infrared spectroscopy, partial least squares discriminant analysis (PLS-DA), and least squares support vector machine (LS-SVM), they could rapidly and effectively identify different concentrations of pesticide residues on cauliflower surfaces [24]. Lapcharoensuk et al. (2023) successfully achieved high-precision detection of chlorantraniliprole pesticide residues in leafy vegetables by combining a portable near-infrared spectrometer with support vector machine (SVM) and principal component analysis neural network (PC-ANN) [25]. Yi et al. (2023) developed a microneedle patch-based surface-enhanced Raman spectroscopy (SERS) sensor that can simultaneously detect pesticide residues on the surface and inside agricultural products. This sensor amplified the Raman signal of pesticide residues efficiently, enabling detection of extremely low concentrations of pesticide residues without damaging the product [26]. Yu et al. (2022) used a multi-scale convolutional neural network model combined with visible/near-infrared spectroscopy technology to achieve high-precision detection of different pesticide residue concentrations on the surface of cantaloupe, with an accuracy rate exceeding 99%. The study indicated that the multi-scale convolutional neural network model has significant advantages in processing complex spectral data, providing new methodological support for pesticide residue detection [27]. Sun et al. (2023) proposed a rapid detection method for pesticide residues on Shanghai bok choy leaves by analyzing near-infrared microscopic images. This method used support vector machine (SVM) and multiple regression analysis to accurately estimate the initial pesticide concentration on the leaves and calculate the time for the pesticide concentration to decrease to acceptable levels based on degradation equations [28].

### 2.3. Application of SERS technology in food pesticide residue detection

Ordinary Raman spectroscopy suffers from weak Raman signals due to low scattering efficiency, which makes it difficult to effectively detect low-concentration substances, thus limiting its applications. In 1974, Fleischmann et al. accidentally discovered that strong Raman signals could be detected when pyridine was adsorbed on the rough surfaces of silver and copper electrodes. They explained that the increased roughness of the electrode surface enhanced the adsorption of pyridine, thereby amplifying the Raman signal [29]. In 1977, Jeanmaire et al. [30] and Albrecht et al. [31] experimentally verified this phenomenon and found that the Raman signal of pyridine on rough metal electrodes was about 106 times stronger than that in solution. This phenomenon is known as surface-enhanced Raman scattering (SERS). In 1997, Kneipp et al. [32] and Nie et al. [33] successfully detected single-molecule Raman signals using SERS technology, solving the problem of weak ordinary Raman signals and low sensitivity, thus driving the widespread development of SERS technology.

SERS technology has found extensive application in the field of food safety, particularly in pesticide detection, and is now capable of detecting multiple pesticide residues. Recently, an increasing number of researchers have focused on the application of SERS technology in pesticide residue detection. Pham et al. successfully applied SERS technology to detect imidacloprid, acephate, and cypermethrin pesticide residues in mangoes by creating a hydrophobic poly(dimethylsiloxane)-modified silver nanoparticle substrate. They developed corresponding quantitative models with detection limits of 0.02 mg/kg, 5 × 10 ⁻ ⁵ mg/kg, and 5 × 10 ⁻ ⁵ mg/kg, enabling rapid detection of pesticide residues in mangoes [34]. Ma et al. synthesized a flexible SERS substrate using gold nanoparticles modified poly(dimethylsiloxane) (PDMS) for in situ detection of methyl parathion pesticide residues on fruit surfaces, with a detection limit of 1.946 μg/mL, without the need for complex sample pretreatment, making it suitable for rapid on-site detection [35]. Liu et al. used Au@Ag core-shell nanorod arrays as substrates and prepared flexible and transparent SERS substrates through a liquid-liquid interface self-assembly method for on-site detection of thiamethoxam pesticide residues on fruits and vegetables. The detection limit of this SERS sensor for thiamethoxam in strawberries, apples, and mushrooms was 2 ng/cm², showing high measurement recovery rates and reproducibility [36]. Chen et al. proposed a filter paper based on PDADMAC/PSS/Au@Ag nanorods, which used SERS technology to rapidly detect nonsystemic pesticide residues on the surfaces of fruits and vegetables. This composite material adsorbed positively charged Au@Ag nanorods onto modified filter paper through electrostatic adsorption, creating three-dimensional SERS hotspots that enabled rapid and sensitive pesticide residue detection [37]. Tao et al. proposed a fiber-optic SERS sensor based on light-wave excitation for efficient detection of thiamethoxam pesticide residues in fruits and vegetables. This sensor successfully achieved a detection limit as low as 10 ⁻ ⁸ M using charge transfer effects, electromagnetic enhancement effects, combined with electrostatic adsorption and laser-induced methods [38].

## 3. Materials and methods

### 3.1. Experimental materials and samples

The glucose standard solution, concentrated sulfuric acid (95.0%–98.0%), chloroauric acid (≥99.9%), acetonitrile (≥99.7%), anhydrous ethanol (≥99.7%), ethyl acetate (≥99.5%), anhydrous sodium sulfate (≥99.0%), as well as high-purity nitrogen or helium gas for gas chromatography analysis, were all purchased from China National Pharmaceutical Group Corporation Chemical Reagents. The solid standards of chlorpyrifos (≥99.9%), pymetrozine (≥99.9%), and o-phenylphenol (OPP, ≥ 99.9%) used in the experiment were all selected for high purity to ensure that spectral detection of target pesticides was not interfered with by impurities. To minimize the impact of solvents on the spectral signals, all experiments were conducted using ultrapure water with a conductivity of 18.25 MΩ·cm to ensure the stability and controllability of the experimental environment.

Fresh apples were selected as the detection matrix for this study. To reduce background interference, all fruit samples were washed multiple times with deionized water and acetonitrile to remove potential surface contaminants and residues. After washing, the samples were air-dried before pesticide coating. A total of 600 μL of different concentrations of chlorpyrifos, pymetrozine, and OPP solutions were evenly applied to the fruit skin surface to ensure uniform pesticide distribution, thereby creating a representative pesticide contamination model. Subsequently, all fruit samples were stored in sealed containers at 4°C in a refrigerator to control the pesticide degradation rate and minimize environmental influences on the detection, thus ensuring the consistency of experimental conditions and enabling the spectral data to accurately reflect the pesticide residue behavior.

### 3.2. Experimental instruments

The main equipment used in this study is listed in Table 1.

**Table 1:** Main Experimental Instruments.

Equipment Name	Model	Manufacturer
Freeze Dryer	FD-1A-50	Labconco Corporation, USA
Desktop Low-Speed Centrifuge	Sorvall ST 8	Thermo Fisher Scientific, USA
Ultrasonic Cleaner	Bransonic M1800	Branson Ultrasonics, USA
Pure Water System	Milli-Q IQ 7000	MilliporeSigma, USA
UV-Vis Spectrophotometer	Cary 60 UV-Vis	Agilent Technologies, USA
Tissue Disruptor	TissueLyser LT	Qiagen, Germany
Near-Infrared Spectrometer	MPA II	Bruker, USA
Mid-Infrared Spectrometer	Antaris II	Thermo Fisher Scientific, USA
FTIR Infrared Spectrometer	Spectrum Two	PerkinElmer, USA
Electronic Analytical Balance	Cubis II	Sartorius, Germany
Portable Raman Spectrometer	XploRA PLUS	Horiba Scientific, Japan
Transmission Electron Microscope	Talos F200i	Thermo Fisher Scientific, USA

### 3.3. Experimental methods

#### 3.3.1. Preparation of pesticide standard solutions.

To comprehensively study the spectral responses of chlorpyrifos, pymetrozine, and o-phenylphenol (OPP) under conventional Raman and SERS conditions, acetonitrile was used as the solvent to prepare seven standard solutions with different concentrations. The concentration gradient covered 10 ⁻ ³ M, 10 ⁻ ⁴ M, 10 ⁻ ⁵ M, 10 ⁻ ⁶ M, 10 ⁻ ^7^ M, and 10 ⁻ ⁸ M. The solutions of chlorpyrifos and pymetrozine were used to explore the sensitivity limitations of conventional Raman detection and the necessity of SERS signal enhancement, while OPP was used as a control group to study the differences in Raman scattering abilities under conventional Raman conditions. Additionally, to investigate the effect of mixed pesticide systems on spectral signals, a 1:1 volume ratio mixture of chlorpyrifos and pymetrozine was prepared to evaluate the spectral changes when these two pesticides are mixed.

#### 3.3.2. Spectral data acquisition.

SERS and NIR spectral techniques were employed to achieve high sensitivity and multi-dimensional data analysis, and to validate the applicability of different detection methods for the target pesticides. For SERS spectral detection, the samples were appropriately diluted and precisely applied to the Q-SERS substrate. Spectral measurements were performed using the RMS1000 portable Raman spectrometer with a laser wavelength set to 785 nm, power fixed at 50 mW, integration time of 2 s, and 3 scan repetitions to obtain high signal-to-noise ratio (SNR) spectral data. NIR spectral detection was carried out using the TS9214 near-infrared spectrometer. After the samples were placed into quartz cuvettes, they were scanned with an integration time of 3 s and 5 scan repetitions to acquire full spectral data.

To ensure scientific rigor and model stability, all spectral data were divided into calibration and prediction sets. For each pesticide concentration, 10 SERS spectra and 10 NIR spectra were collected, totaling 50 SERS spectra and 50 NIR spectra. Six spectra were randomly selected to form the calibration set for building the PLSR (Partial Least Squares Regression) quantitative model, ensuring accurate fitting of pesticide signals at different concentrations. The remaining four spectra were assigned to the prediction set to assess the model’s generalization ability and test the accuracy of pesticide residue detection. In total, the calibration set included 36 SERS spectra and 36 NIR spectra, while the prediction set included 24 SERS spectra and 24 NIR spectra, ensuring balanced data for reliable model development.

### 3.4. Spectral data preprocessing

During the acquisition of NIR and SERS spectra, interference from instrument noise, baseline drift, and light scattering is inevitable. Therefore, preprocessing of spectral data is crucial to improving the accuracy of subsequent analysis. It is especially important to note that the spectral intensity of NIR and SERS spectra differs significantly, resulting in them being at different magnitudes. To effectively combine the spectral information, both spectral datasets were normalized, scaling the spectral intensity to the same level. The following spectral data preprocessing methods were used in this study: (1) baseline correction was performed using Adaptive Iterative Re-weighted Penalized Least Squares (AIRPLS); (2) Multi-Scatter Correction (MSC) was applied for light scattering correction; (3) spectral smoothing was performed using the Savitzky-Golay convolution smoothing algorithm; (4) Min-Max normalization was used to scale the spectral intensity between 0 and 1.

### 3.5. Spectral data fusion

**Fig 1:**
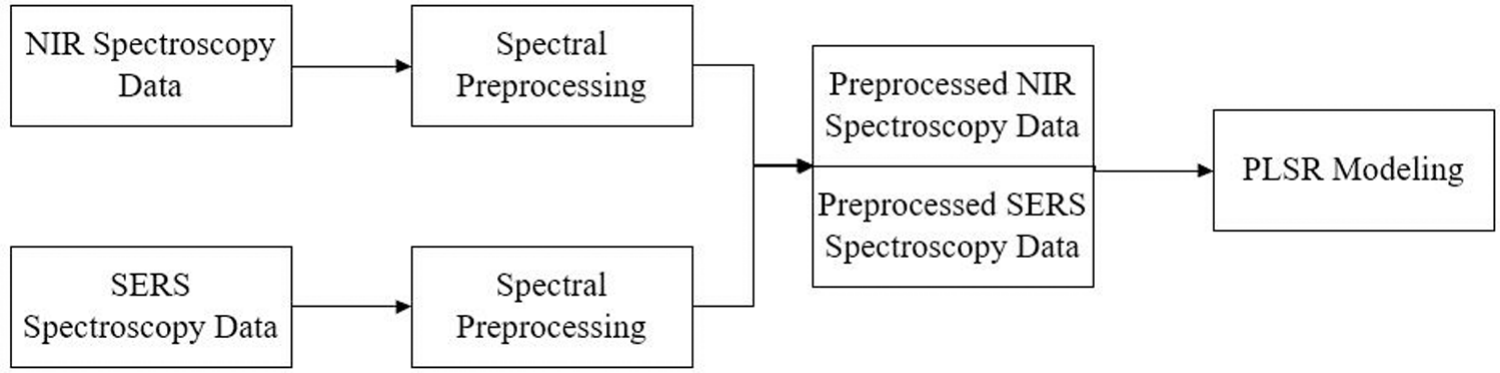
Schematic diagram of the direct fusion process for spectral data.

Direct fusion of spectral data is the simplest and most direct fusion method. It involves combining data from different spectral sources in a specified order to form a matrix, based on which a qualitative or quantitative model (in this study, a PLSR model) is constructed to enhance the prediction ability of the model. It is important to emphasize that preprocessing of the original spectral data is required before fusion. Fig 1 illustrates the process flow for direct fusion of spectral data.

**Fig 2:**
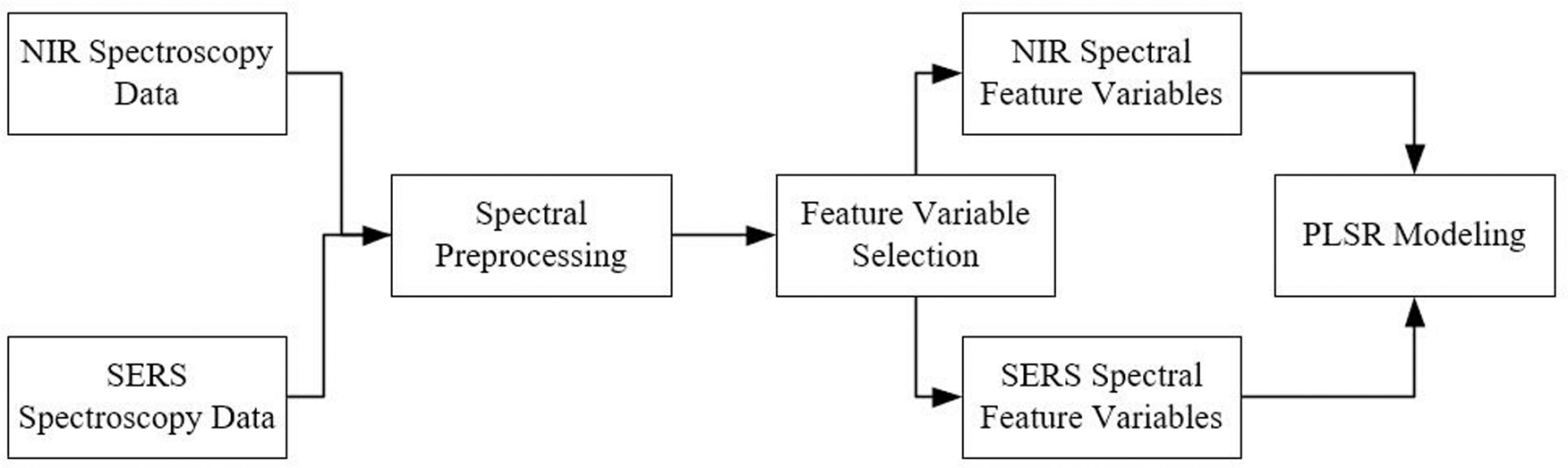
Schematic diagram of the feature-level fusion process for spectral data.

Feature-level fusion of spectral data involves the integration of original spectral data at the feature information level. Specifically, after preprocessing the data from different spectral sources, the feature variables are selected and arranged in a specified order to form a matrix. Based on this matrix, a qualitative or quantitative model (PLSR in this study) is constructed. Since original spectral data contains a large number of redundant variables, feature-level fusion typically performs better than direct fusion. Fig 2 shows the process flow for feature-level fusion of spectral data.

### 3.6. Spectral feature variable selection

The HSIC-VSIO algorithm chosen for this study to perform spectral feature variable selection is described in the following main steps:

The first step involves the use of the Weighted Binary Matrix Sampling (WBMS) strategy to collect variable samples. The WBMS strategy is an advanced sampling method developed based on the Binary Matrix Sampling (BMS) strategy. During the sampling process, this strategy assigns different numbers of “0”s and “1”s to each column of variables according to the weight differences, with higher weight values being more likely to be selected.

The second step is to perform independence testing using the Hilbert-Schmidt Independence Criterion (HSIC). The HSIC criterion is a novel criterion developed based on the Reproducing Kernel Hilbert Space (RKHS). Suppose two random variables, X and Y, and the RKHS of the nonlinear feature mapping is set as F(ϕ:X→F ). If there exists x∈X, then there must exist ϕ(x)∈F. This situation applies to the same setup of G(ϕ:Y→G), and the cross-covariance operator expression between F and G can be set as follows:


$$C_{xy}=E_{x,y}[(\emptyset (x)-\mu_{\text{x}})⊗(\psi (y)-\mu_{\text{y}})]$$ (1)

In the formula, $\mu_{x}=E_{x}\left[\Phi \left(x\right)\right]$; $\mu_{y}=E_{y}\left[\psi \left(y\right)\right]$; $E_{x,y}$ represent the expectation of the joint probability density function $P_{xy}$; $E_{x}$ and $E_{y}$ represent the expectations of the marginal probability density functions $P_{x}$ and $P_{y}$, respectively;  ⊗  is the operator for tensor product computation.

The theoretical value of HSIC can be expressed as the square of the Hilbert-Schmidt norm of $C_{xy}$, as shown in the following expression:


$$\begin{array}{c} \text{HSIC}(X,Y)=∥C_{\text{xy}HS}^{2}∥=E_{\text{x},{\text{x}}^{\prime },\text{y},{\text{y}}^{\prime }}[k(x,x^{\prime })l(y,y^{\prime })]+\\ \begin{array}{l} E_{\text{x},{\text{x}}^{\prime }}[k(x,x^{\prime })]E_{\text{y},{\text{y}}^{\prime }}[l(y,y^{\prime })]\\ -2E_{\left(\text{x},\text{y}\right)}[E_{{\text{x}}^{\prime }}[k(x,x^{\prime })]E_{(}y^{\prime })[l(y,y^{\prime })]] \end{array} \end{array}$$ (2)

In the formula, $k(x,x^{\prime })=<\phi (x),\text{Φ}(x^{\prime })>$; $l(y,y^{\prime })=<\text{Ψ}(y),\text{Ψ}(y^{\prime })>$; k, l are kernel functions (k: X×X→R; I:Y×Y→R); $E_{\text{x},{\text{x}}^{\prime },\text{y},{\text{y}}^{\prime }}$ represents the joint expectation of data pairs (x,y) and $\left(x^{\prime },y^{\prime }\right)$.

Suppose the number of independent observations is n, then there exists $Z=\{(x_{1},y_{1})\ldots (x_{n},y_{n})\}\subset (X\times Y)$, and its empirical value expression is as follows:


$$\text{HSIC}(Z,F,G)=\frac{1}{{\left(n-1\right)}^{2}}tr(KHLH)$$ (3)

In the formula, $K,H,L\in R^{n\times n}$; $K_{\text{ij}}=k(x_{\text{i}},x_{\text{j}})$; $L_{\text{ij}}=l(y_{\text{i}},y_{\text{j}})$; $H_{\text{ij}}=L-N^{-1}ee^{\text{T}}$, where e is the vector of all ones, and _*tr* represents the trace of the matrix KHLH._

If the random variables are independent, the value of their HSIC is 0. Therefore, HSIC is suitable for independence testing.

The third step is to assess the importance of wavelength variables. Let X represent the spectral data matrix and y represent the response vector. The expression for the full-spectrum multivariate calibration model is as follows:


$$y=2f_{\text{full}}(x_{1},...,x_{\text{p}})+\varepsilon_{\text{full}}$$ (4)

In the formula, $f_{\text{full}}$ represents the full-spectrum multivariate calibration model established by _PLSR_, and $\varepsilon_{\text{full}}$ represents the prediction residuals.

By removing the m-th wavelength, a new multivariate calibration model can be constructed, expressed as:


$$y={\hat{f}}_{\text{m}}(x_{1},...,x_{\text{m}-1},x_{\text{m}+1},...,x_{\text{p}})+\varepsilon_{\text{m}}$$ (5)

In the formula, ${\hat{f}}_{\text{m}}$ represents the multivariate calibration model established by PLSR, and ${\hat{f}}_{\text{m}}$ represents the prediction residuals, which contain noise information as well as information from the wavelength variable $x_{\text{m}}$.

If the statistical independence difference between the prediction residuals and the outputs can be obtained, the importance of the wavelength can be reasonably inferred, as expressed in the following formula:


$$WI_{\text{m}}=\frac{\text{HSIC}(y,\varepsilon_{\text{m}})}{\text{HSIC}(y,\varepsilon_{\text{full}})}\;$$ (6)

In the formula, $f_{\text{full}}$ represents the importance of wavelength variable $x_{\text{m}}$, where m=1,2,....,p.

The importance of all wavelength variables is calculated and normalized. The values before and after normalization are stored in vectors $T=[WI_{1},WI_{2},\ldots ,WI_{\text{p}}]$ and $T^{\prime }=[WI_{1}^{\prime },WI_{2}^{\prime },...,WI_{p}^{\prime }]$, respectively.

The fourth step is based on WBMS (with the size of the binary matrix being M×p). The initial weight values of the wavelength variables are set to 0.5. M multivariate calibration models established by PLSR are obtained, and their 5-fold cross-validation root mean square errors (RMSECV) are computed. The model with the minimum value of RMSECV is identified, and its proportion is set to *σ*, with its value marked as minRMSECV.

At the same time, the frequency of each wavelength variable appearing in the model is calculated $f_{\text{i}}$, expressed as:


$$f_{\text{i}}=\frac{N_{\text{i}}}{M\times \sigma }$$ (7)

In the formula, N_i_ is the number of times the i-th wavelength variable appears in the model.

The frequency $f_{\text{i}}$ of the wavelength variables is stored in vector $Q=[f_{1},f_{2},\ldots ,f_{\text{p}}]$, and the weights of the wavelength variables are updated, $W=0.5\times T^{\prime }+0.5\times Q$.

The fifth step, based on the updated *W* and WBMS strategy, involves constructing M new multivariate calibration models using PLSR. Similar to the fourth step, the model corresponding to minRMSECV is found. If ${\text{minRMSECV}}_{\text{I}}<{\text{minRMSECV}}_{\text{I}-1}$, where I represents the iteration number, the frequencies of the wavelength variables need to be recalculated, and the subsequent operations should be repeated until ${\text{minRMSECV}}_{\text{I}}<{\text{minRMSECV}}_{\text{I}-1}$. At this point, the iteration ends, and the optimal wavelength variables are output.

Based on the research of existing scholars, the main parameters set for the HSIC-VSIO in this study are as follows: the number of rows in the binary matrix is 1000, *σ* is 10%, and the rationality of the parameter settings will be verified later.

### 3.7. Model evaluation metrics

To assess the performance of each model, a series of evaluation metrics are typically used. The specific evaluation metrics are shown in Table 2:

**Table 2:** Model Evaluation Metrics.

Metric Name	Expression
Calibration Set RMSE	*RMSE* ^1^
Prediction Set RMSE	*RMSE* ^2^
Calibration Set R²	$R^{2}$
Prediction Set R²	$R^{2'}$
Performance Deviation Ratio	*RPD*

The specific formulas for these evaluation metrics are as follows:


$$\text{RMSE}=\sqrt{\frac{1}{L}\sum_{i=1}^{L}{\left(y_{\text{i}}-{\hat{y}}_{\text{i}}\right)}^{2}}$$ (8)

$$R^{2}=1-\frac{\sum_{i=1}^{L}{\left(y_{\text{i}}-{\hat{y}}_{\text{i}}\right)}^{2}}{\sum_{L}^{i=1}{\left(y_{\text{i}}-\bar{y}\right)}^{2}}$$ (9)

$$\text{RPD}=\frac{1}{\sqrt{1-R_{p}^{2}}}$$ (10)

In the formula, $y_{\text{i}}$, ${\hat{y}}_{\text{i}}$, and $\bar{y}$ represent the true values, predicted values, and the mean of the true values, respectively.

## 4. Results and analysis

### 4.1. Spectral data analysis

**Fig 3:**
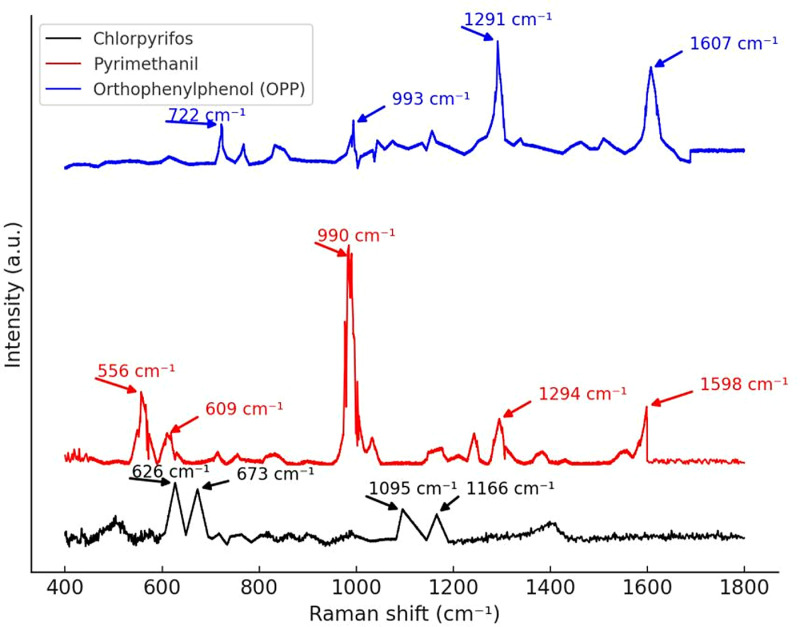
Raman spectra.

The solid-state Raman spectra of chlorpyrifos, pymetrozine, and o-phenylphenol (OPP) are shown in Fig 3, please refer to S1 Data for the data. It is evident that these three pesticides exhibit clear and stable Raman characteristic peaks without solvent interference. The main Raman characteristic peaks of chlorpyrifos are located at 627 cm ⁻ ¹, 673 cm ⁻ ¹, 1095 cm ⁻ ¹, and 1166 cm ⁻ ¹. These peaks correspond to the C-Cl stretching vibration, P = S stretching vibration, and the aromatic ring skeletal vibration, indicating that chlorpyrifos has distinct Raman vibrational modes in the solid state. The main Raman characteristic peaks of pymetrozine are located at 556 cm ⁻ ¹, 609 cm ⁻ ¹, 990 cm ⁻ ¹, 1294 cm ⁻ ¹, and 1598 cm ⁻ ¹, while the main peaks of OPP are located at 722 cm ⁻ ¹, 993 cm ⁻ ¹, 1291 cm ⁻ ¹, and 1607 cm ⁻ ¹. The differences in these peaks are significant in the solid state, suggesting that the molecular structure and vibrational modes of different pesticides are unique, with their Raman activity being ideal in the absence of solvent interference. However, solid-state spectra only provide fundamental molecular information, and since pesticides are typically found in solution form in real-world applications, it is crucial to investigate their spectral behavior in solution.

As OPP is also used in food processing and storage, studying its Raman spectrum helps to explore the differences in the performance of various pesticide molecules under conventional Raman conditions. However, the primary goal of this study is to detect mixed chlorpyrifos and pymetrozine residues. Therefore, in subsequent experiments, OPP was primarily used as a comparison to examine the Raman scattering ability of different molecules under conventional Raman conditions to further clarify the detectability of various pesticides.

**Fig 4:**
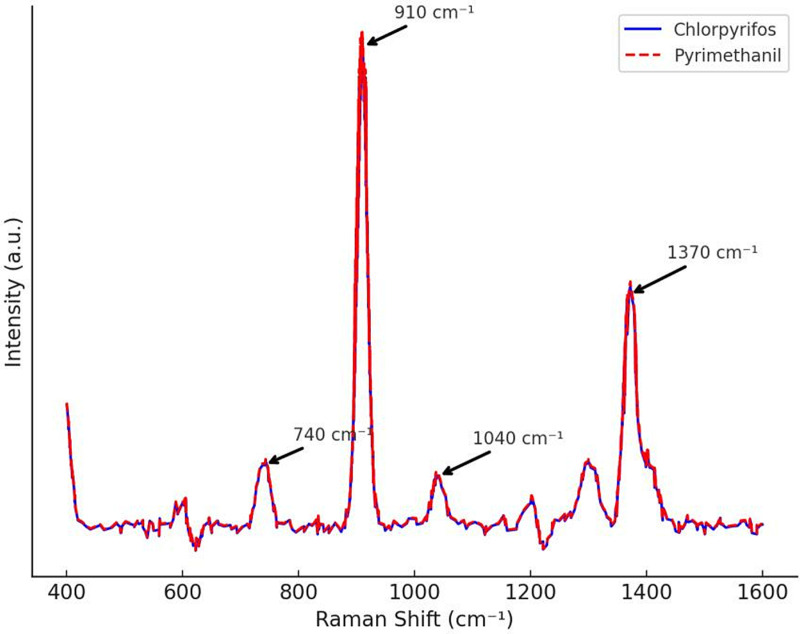
Conventional Raman spectra of chlorpyrifos and pymetrozine solutions.

By comparing the Raman spectra of chlorpyrifos and pymetrozine solutions at a concentration of 10 ⁻ ³ M without the addition of gold nanoparticles (shown in Fig 4), it can be observed that no characteristic peaks of the two pesticides appear in the spectra. Instead, peaks at 740 cm ⁻ ¹, 910 cm ⁻ ¹, 1040 cm ⁻ ¹, and 1370 cm ⁻ ¹ correspond to solvent peaks, which do not overlap with the pesticide’s characteristic peaks, please refer to S2 Data for the data. Compared to the solid-state spectra, the Raman signals of chlorpyrifos and pymetrozine in solution are significantly weakened or even disappear. This phenomenon suggests that under conventional Raman conditions, the molecular scattering ability of chlorpyrifos and pymetrozine is relatively low, and their Raman signals are shielded or overwhelmed by the solvent environment, making it difficult for conventional detection methods to directly capture their molecular vibration modes. The shielding effect of the solvent on the Raman signal may stem from the dipole-dipole interactions between solvent molecules and pesticide molecules, which weakens the optical response of the molecules themselves. Moreover, the smaller Raman scattering cross-section of chlorpyrifos and pymetrozine further limits the spectral signal, making it difficult to overcome background noise under conventional Raman conditions, thus reducing the feasibility of detection.

**Fig 5:**
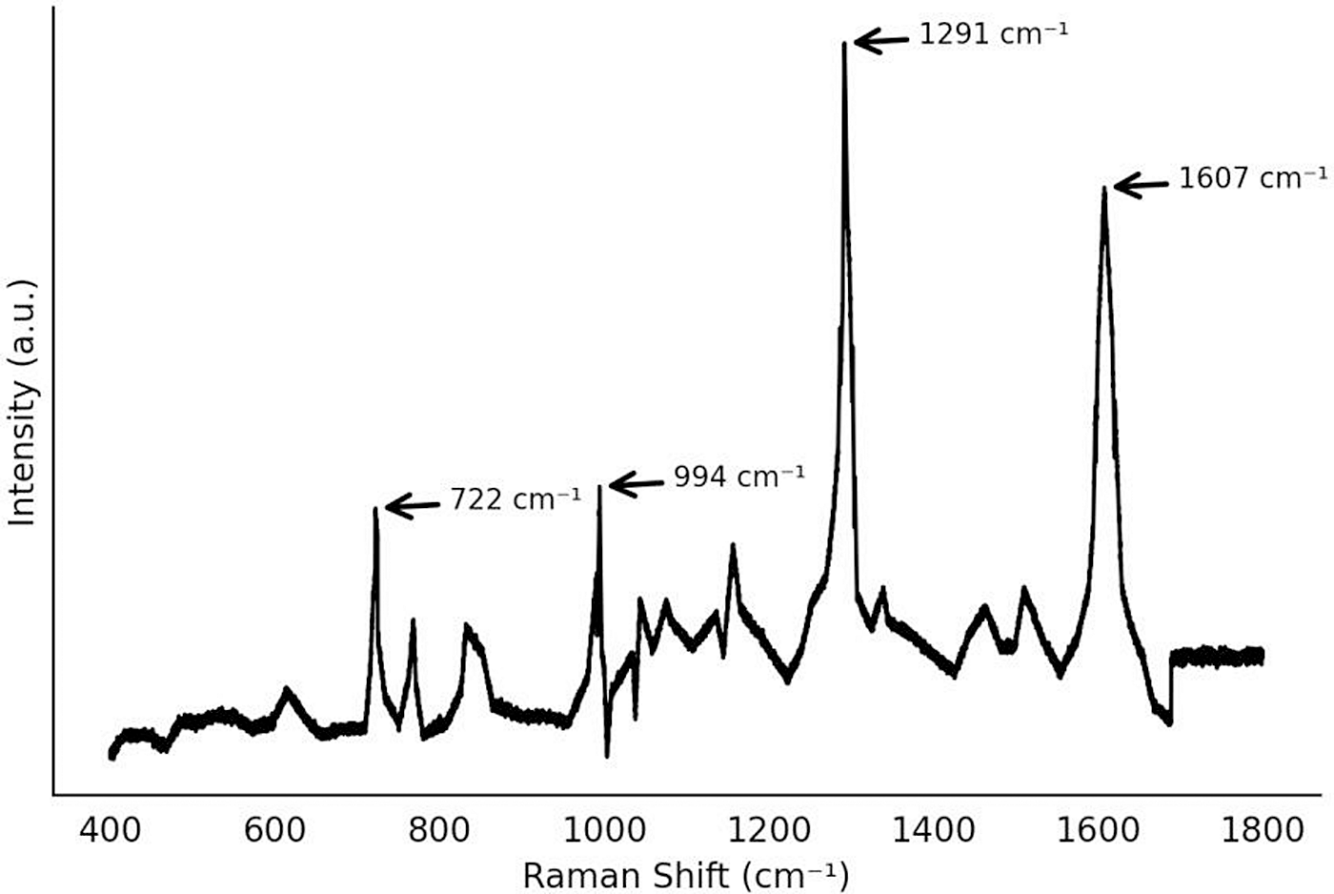
Conventional Raman spectra of OPP solution.

To further investigate the signal differences between different pesticide molecules under conventional Raman conditions, this study also conducted conventional Raman spectral tests on an OPP solution (Fig 5), please refer to S3 Data for the data. In contrast to the Raman spectra of chlorpyrifos and pymetrozine, OPP still exhibits distinct Raman signals, although the peak positions have slightly shifted compared to its solid-state spectrum. This shift may be due to solvation effects of OPP in the solution, leading to changes in the molecular environment and causing energy shifts in some of the vibrational modes.

From the detection perspective, the spectral behavior of chlorpyrifos and pymetrozine solutions indicates that conventional Raman spectroscopy alone cannot achieve high-sensitivity pesticide detection. Therefore, signal enhancement techniques must be introduced to improve the detectability of molecular scattering signals. In contrast, o-phenylphenol (OPP) exhibits stronger Raman scattering ability, and its solution spectrum can still be detected under conventional Raman conditions. However, this does not imply that all pesticides are suitable for conventional Raman detection. The detectability of Raman signals depends not only on the Raman scattering cross-section of the molecules but also on factors such as the solvent environment, molecular structural characteristics, and optical response. Therefore, to achieve high-sensitivity detection of target pesticides, this study further introduces Surface-Enhanced Raman Spectroscopy (SERS) technology. By using gold nanoparticles as the enhancement substrate, the localized surface plasmon resonance (LSPR) effect significantly amplifies the Raman signal, allowing pesticide molecules with small Raman scattering cross-sections to still be effectively detected.

**Fig 6:**
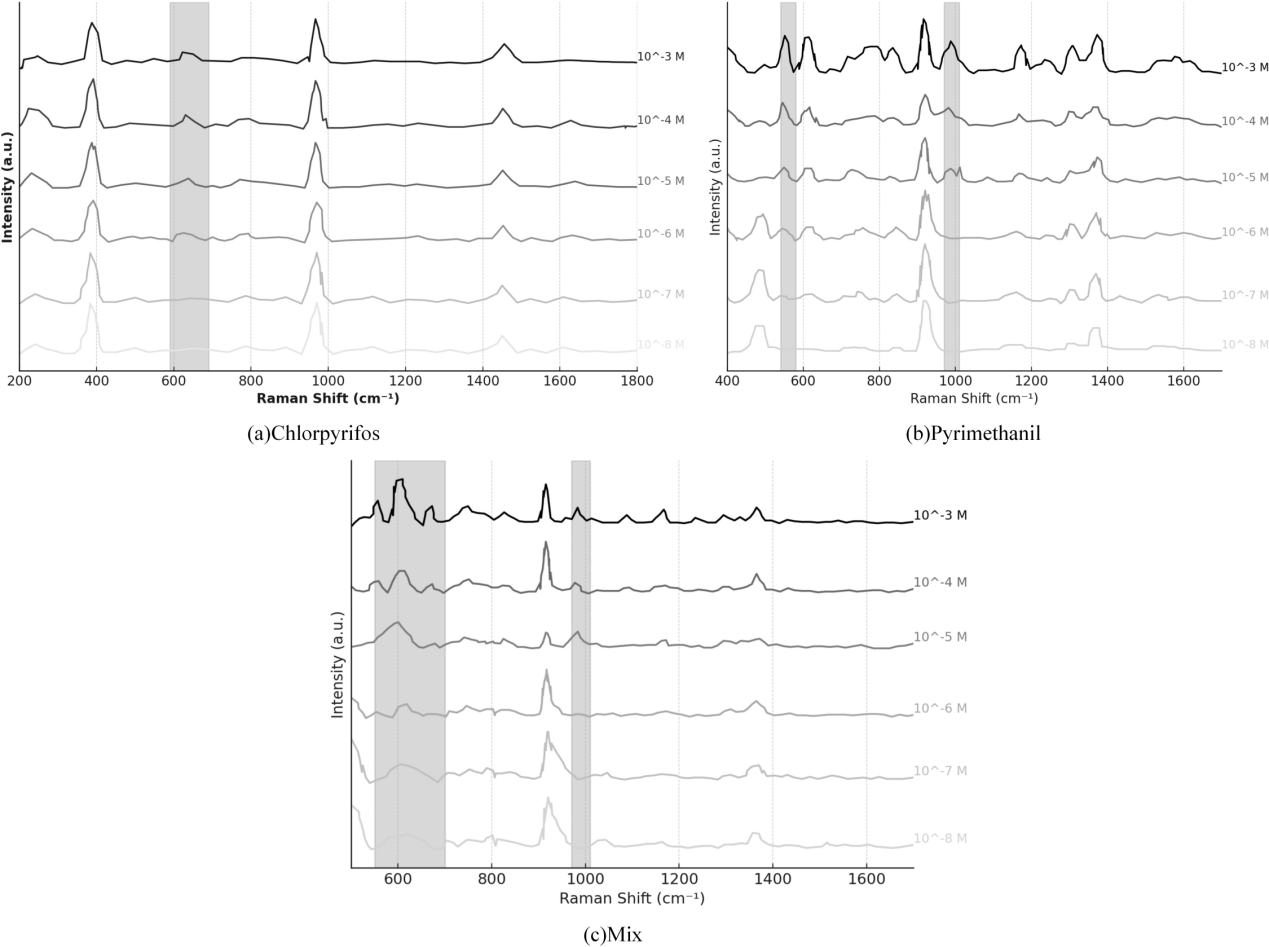
SERS spectra of standard solutions of chlorpyrifos, pymetrozine, and their equimolar mixture.

On this basis, this study detected standard solutions of chlorpyrifos, pymetrozine, and their equimolar mixture, and the results are shown in Fig 6, please refer to S4 Data for the data. As seen in Fig 6, the standard solutions of chlorpyrifos and pymetrozine exhibit clear Raman characteristic peaks over different wavenumber ranges. Even after mixing the two pesticides, these characteristic peaks are well retained in the SERS spectrum, demonstrating the sensitivity and specificity of SERS technology in pesticide residue detection. In the SERS spectrum of chlorpyrifos solution, the most prominent Raman characteristic peaks are observed at 609 cm ⁻ ¹ and 990 cm ⁻ ¹, which are highly correlated with the vibrational modes of the C-Cl and P = S bonds in the chlorpyrifos molecular structure. For pymetrozine, the SERS spectrum exhibits significant signal enhancement at 558 cm ⁻ ¹ and 990 cm ⁻ ¹. The peak at 558 cm ⁻ ¹ can be attributed to the stretching vibration of the C-N bond, while the 990 cm ⁻ ¹ peak likely corresponds to the characteristic vibration of the cyclic molecular structure. When chlorpyrifos and pymetrozine are mixed in an equimolar ratio, the SERS spectrum displays the characteristics of a two-component superposition. However, it is worth noting that some peak positions show slight frequency shifts, which may result from chemical enhancement effects due to molecular interactions. Notably, the peaks at 558 cm ⁻ ¹ and 990 cm ⁻ ¹ still maintain high intensity in the mixed spectrum, further confirming the signal independence of chlorpyrifos and pymetrozine in SERS detection. This ensures that the characteristic peaks of the two pesticides do not significantly overlap, making the method feasible for detecting mixed pesticide systems. However, as the concentration decreases further (to 10 ⁻ ⁶ M and below), the SERS characteristic peak intensities of both pesticides decrease significantly, especially at 10 ⁻ ^7^ M, where their characteristic peaks nearly disappear. This suggests that pesticide residues at these low concentrations are no longer effectively detectable. This phenomenon indicates that when pesticide residue levels are extremely low, the SERS signal is increasingly affected by background noise, leading to a decline in signal detectability. It is noteworthy that within the 10 ⁻ ⁶ M concentration range, the characteristic peaks of chlorpyrifos at 609 cm ⁻ ¹ and pymetrozine at 559 cm ⁻ ¹ are still detectable but with lower signal intensities and are close to background noise, showing that the SERS technology is approaching its detection limit at this concentration. Moreover, the spectra of the mixed chlorpyrifos and pymetrozine solution show that at high concentrations, the characteristic peaks of both pesticides coexist without significant interference. However, at low concentrations, some peaks experience attenuation or even disappearance. This indicates that in practical detection applications, the mixed residues of pesticides may further affect the signal enhancement effect, thus reducing detection accuracy. Therefore, the NIR-SERS combined analytical method proposed in this study can not only achieve high-sensitivity recognition of pesticides using SERS but also evaluate the overall chemical composition using NIR, helping to improve the detection accuracy of low-concentration pesticides.

### 4.2. Verification results of parameter setting rationality

To verify the rationality of the HSIC-VSIO algorithm parameter settings, this study performed feature variable selection on NIR and SERS spectral data under different parameter settings. The selected feature variables were then fused to construct PLSR models. This study deeply analyzes the feature selection ratio (σ), the number of feature variables (K), spectral data preprocessing methods, and PLSR dimensionality (L) to ensure optimal model performance and maximized computational efficiency.

In the feature selection process, the parameter σ directly determines the proportion of feature variables entering the model. If σ is too large, redundant noise may be introduced; if it is too small, key information may be lost. Therefore, this study sets σ =  10%, 20%, 30%, and 40% for comparative analysis, examining their effects on the model’s fitting ability (RMSEC, $R_{c}^{2}$) and prediction ability (RMSEP, $R_{p}^{2}$, RPD).

From Table 3, it is visually observed that when σ =  10%, both the RMSEC and RMSEP reach their minimum, while $R_{c}^{2}$ and $R_{p}^{2}$ approach 1, and RPD also achieves the highest value. This indicates that when the feature variable selection ratio is 10%, the model retains key information while effectively suppressing noise interference, making both fitting and prediction optimal. In contrast, as the σ value increases (20% and above), RMSEC and RMSEP show an increasing trend, and $R_{c}^{2}$ and $R_{p}^{2}$ gradually decrease, with RPD also declining. This phenomenon suggests that when too many features are introduced, the model is affected by redundant variables, leading to a decline in fitting ability and weakened generalization ability. Therefore, from an overall performance perspective, setting σ =  10% effectively reduces feature redundancy, improves model accuracy, and ensures computational efficiency, making it the optimal feature selection ratio in this study.

**Table 3:** Effect of Parameter σ on Model Prediction Performance.

σ(%)	RMSEC	$R_{c}^{2}$	RMSEP	$R_{p}^{2}$	RPD
10	0.1571	0.9920	0.1832	0.9878	8.2758
20	0.1783	0.9812	0.1986	0.9734	7.1269
30	0.2015	0.9724	0.2203	0.9652	6.2869
40	0.2180	0.9588	0.2035	0.9763	6.1799

In high-dimensional spectral data processing, the number of feature variables (K) directly determines the amount of information in the model and the computational burden. Too few features may lead to information loss and affect prediction accuracy, while too many features may introduce noise, resulting in increased model complexity and decreased generalization ability. Therefore, we set K =  10, 20, 30, and 40 for analysis to evaluate the optimal number of feature variables.

From Table 4, it can be seen that when K =  20, both RMSEC and RMSEP reach their lowest points, while $R_{c}^{2}$ and $R_{p}^{2}$ reach their peak values, and RPD is also optimal. This suggests that when the number of features is 20, the model achieves the best balance between information utilization and computational efficiency. When K is less than 20 (i.e., K =  10), insufficient information extraction causes RMSEC and RMSEP to increase significantly, weakening the model’s prediction ability. When K exceeds 20 (i.e., K =  30, 40), although the information amount increases, noise also rises, leading to overfitting and reduced generalization ability. Therefore, K =  20 is the optimal choice, as it maximizes the balance between information extraction and model complexity, ensuring the dual optimization of prediction accuracy and computational efficiency.

**Table 4:** Effect of Parameter K on Model Prediction Performance.

K	RMSEC	$R_{c}^{2}$	RMSEP	$R_{p}^{2}$	RPD
10	0.1893	0.9805	0.2024	0.9756	7.4821
20	0.1574	0.9928	0.1815	0.9883	8.3124
30	0.1752	0.9837	0.1957	0.9748	7.1645
40	0.1981	0.9721	0.2179	0.9613	6.7428

Next, the effect of spectral data quality needs to be considered. The quality of spectral data directly affects the stability and prediction accuracy of the model. Raw spectral signals are often interfered with by various factors such as environmental noise, baseline drift, and light scattering effects. These noises not only mask important feature information but may also lead the model to learn non-essential variables, thus affecting the final prediction ability. Therefore, selecting appropriate spectral preprocessing methods is a key step in improving the signal-to-noise ratio and optimizing data quality. This study compared three common spectral preprocessing methods to assess their effects on model performance. The Savitzky-Golay (SG) filter is a widely used smoothing technique that reduces high-frequency noise while retaining as much spectral detail as possible. SG +  SNV (Standard Normal Variate) further normalizes the data distribution on top of SG filtering, eliminating baseline drift and making the signal more stable. SG +  SNV +  MSC (Multiplicative Scatter Correction) additionally corrects for scattering effects based on the first two methods, enhancing feature signals and improving data consistency and comparability.

The results in Table 5 show that different spectral preprocessing methods significantly impact the model’s fitting ability, generalization performance, and stability. When only using SG filtering, although it can reduce high-frequency noise to some extent, RMSEC and RMSEP remain relatively high, indicating that spectral data still contain significant interference, affecting the model’s learning effectiveness. However, after adding SNV normalization on top of SG filtering, both RMSEC and RMSEP decreased noticeably, and both $R_{c}^{2}$ and $R_{p}^{2}$ were significantly improved. This suggests that this method effectively reduces baseline drift, stabilizing the spectral signal and enhancing the model’s prediction ability. When MSC is further introduced for scattering correction, the model performance reaches its peak. At this point, RMSEC and RMSEP reach their minimum values, $R_{c}^{2}$ and $R_{p}^{2}$ approach 1, and RPD reaches the highest level. This indicates that this preprocessing strategy not only reduces scattering interference to the greatest extent but also significantly improves the model’s accuracy and robustness. The optimized data are more suitable for high-precision modeling. Therefore, considering data quality, model accuracy, and computational efficiency, this study ultimately selects SG +  SNV +  MSC as the optimal spectral preprocessing method to ensure high-quality input for spectral data, thereby enhancing the model’s recognition ability and generalization performance.

**Table 5:** Effect of Different Preprocessing Methods on Model Prediction Performance.

Preprocessing Method	RMSEC	$R_{c}^{2}$	RMSEP	$R_{p}^{2}$	RPD
SG	0.1782	0.9815	0.2019	0.9742	7.2364
SG+SNV	0.1594	0.9902	0.1847	0.9856	8.1473
SG+SNV+MSC	0.1489	0.9945	0.1762	0.9901	8.6347

Finally, regarding the selection of PLSR dimensionality (L), L affects both the model’s ability to extract information and the computational complexity and generalization performance. To find the optimal dimensionality, we conducted systematic experiments with L =  5, 10, 15, and 20 to investigate the specific impact of different L values on model performance.

The results in Table 6 indicate that when L =  10, the model achieves the minimum values for both RMSEC and RMSEP, $R_{c}^{2}$ and $R_{p}^{2}$ reach their maximum values, and RPD is at its best state. This suggests that, at this dimensionality, the model effectively extracts information while avoiding noise interference, achieving the optimal fitting and prediction ability. In contrast, when L =  5, the model’s prediction accuracy declines due to insufficient information extraction, reflected in the increased RMSEC and RMSEP and decreased coefficient of determination, indicating that this dimensionality is not sufficient to capture key information in the data. On the other hand, when L is too large (i.e., L =  15 or 20), RMSEC and RMSEP increase, while $R_{c}^{2}$ and $R_{p}^{2}$ decrease, indicating that as the dimensionality increases, the model becomes interfered with by redundant information, leading to a decrease in generalization ability. From the table data, it is clear that appropriately setting the PLSR dimensionality is crucial for improving model performance. When L =  5, the limited information extraction leads to poor model performance; when L =  15 or above, the introduction of redundant features not only increases computational load but also reduces prediction ability, affecting the model’s stability. Considering model accuracy, generalization ability, and computational cost, this study ultimately selects L =  10 as the optimal PLSR dimensionality to ensure that the model can adequately learn data features while avoiding overfitting, thus achieving the best prediction results.

**Table 6:** Effect of Parameter L on Model Prediction Performance.

L	RMSEC	$R_{c}^{2}$	RMSEP	$R_{p}^{2}$	RPD
5	0.1903	0.9792	0.2048	0.9735	7.2984
10	0.1495	0.9938	0.1759	0.9905	8.5243
15	0.1687	0.9851	0.1892	0.9783	7.8451
20	0.1856	0.9763	0.2057	0.9689	7.4267

### 4.3 Comparison of Method Effectiveness

To further assess the fusion capability of SERS and NIR spectral data, as well as the signal variations of mixed pesticide samples in SERS detection, this study conducted PLSR modeling using NIR spectral data, SERS spectral data, direct fusion of NIR and SERS data, and feature-level fusion of NIR and SERS data. The performance comparison was based on data from mixed pesticide samples of chlorpyrifos and pyrimethanil. During the PLSR modeling process, a 5-fold cross-validation was employed to determine the optimal number of latent variables (LVs), and key performance metrics such as the root mean square errors of calibration (RMSE1), prediction (RMSE2), coefficient of determination (R²), and relative error ratio (RPD) were recorded to evaluate the prediction capabilities of the different methods. Table 7 presents the experimental results of different methods in mixed pesticide detection.

**Table 7:** Results of Different Methods in Mixed Pesticide Detection.

Detection Method	LVs	Calibration Set		*Prediction Set*		
		RMSE1	$R^{2}$	RMSE2	$R^{2'}$	RPD
NIR	10	0.285	0.956	0.350	0.920	3.500
SERS	8	0.214	0.976	0.238	0.972	5.650
NIR + SERS (Direct Fusion)	10	0.195	0.986	0.215	0.974	5.820
NIR + SERS (Feature-Level Fusion)	9	0.160	0.994	0.185	0.988	8.290

The results in Table 7 show that significant differences exist between the various spectral modeling methods for both calibration and prediction sets. First, the optimal number of latent variables for the NIR spectral data model was 10, with a calibration RMSE of 0.285, a calibration *R²* of 0.956, a prediction RMSE of 0.350, a prediction *R²* of 0.920, and an RPD of only 3.500. These results indicate that while the NIR spectral data model possesses some prediction capability in pesticide residue detection, its relatively low RPD value suggests that the model has weaker robustness and generalization ability, making it difficult to maintain high precision in complex environments. In contrast, the SERS spectral data model outperformed the NIR model. The optimal number of latent variables for this model was 8, with a calibration RMSE of 0.214, a calibration *R²* of 0.976, a prediction RMSE of 0.238, a prediction *R²* of 0.972, and an RPD of 5.650. Compared to the NIR model, the SERS spectral data model showed significantly reduced errors in both the calibration and prediction sets, higher *R²* values, and a notable increase in RPD, indicating that this method is not only more accurate but also more robust and capable of generalizing to a wider range of complex sample detections. When analyzing the fusion strategies for NIR and SERS spectral data, the direct fusion method yielded an optimal number of latent variables of 10. The calibration RMSE dropped to 0.195, the calibration *R²* increased to 0.986, the prediction RMSE further decreased to 0.215, and the prediction *R²* reached 0.974, with an RPD value increasing to 5.820. These results suggest that the direct fusion method effectively enhanced the model’s fitting ability and prediction accuracy compared to individual NIR or SERS spectral data, allowing the model to demonstrate better adaptability for more complex pesticide residue detection tasks.

However, the best performance was achieved with the feature-level fusion method of NIR and SERS. The optimal number of latent variables was 9, with the calibration RMSE further reduced to 0.160, the calibration *R²* increased to 0.994, the prediction RMSE decreased to 0.185, the prediction *R²* reached 0.988, and the RPD value peaked at 8.290, significantly outperforming the other methods. Overall, the feature-level fusion method not only exhibited the lowest errors in both calibration and prediction sets but also achieved the highest *R²* and a substantial improvement in RPD. This indicates that this method can maximally extract complementary information from both NIR and SERS spectral data, improving the model’s generalization ability and making pesticide residue detection more precise and reliable.

During the experiment, we further explored the signal response of SERS in the detection of chlorpyrifos and pyrimethanil at low concentrations. The experimental results showed that even at a concentration of 10 ⁻ ⁶ M in the mixed solution, the SERS characteristic peaks at 609 cm ⁻ ¹ for chlorpyrifos and 558 cm ⁻ ¹ for pyrimethanil were still visible. However, the signal intensity was significantly weaker compared to higher concentration samples, and as the concentration further decreased, the noise level increased, reducing the detectability of the signals. When the pesticide concentration dropped to 10 ⁻ ^7^ M, the characteristic peak signals approached baseline levels, indicating that SERS had reached its detection sensitivity limit at this concentration. Further calculations revealed that the detection limits for chlorpyrifos on apple peel were 2.1 × 10 ⁻ ⁴ mg/cm², and for pyrimethanil, they were 3.5 × 10 ⁻ ⁴ mg/cm², indicating that SERS still provided a strong signal response even at low concentrations, with slightly higher sensitivity for chlorpyrifos than for pyrimethanil.

To investigate the impact of the apple peel matrix on spectral signals, this study also compared NIR and SERS data features. The experiment revealed that the main characteristic peaks of the SERS spectrum did not shift significantly, suggesting that the apple matrix did not cause significant interference with the chemical enhancement effect of SERS. However, in the NIR spectrum, the moisture content of the apple peel influenced the spectral signal, leading to a slight enhancement of the absorption peak around 1450 cm ⁻ ¹, reflecting the unique absorption characteristics of fruit matrices in near-infrared spectra. Further analysis indicated that in the NIR +  SERS direct fusion model, the effect of the apple matrix on the prediction results was more noticeable, while in the feature-level fusion model, this influence was significantly suppressed. This is because the feature-level fusion method effectively extracted key spectral information while minimizing background interference from the apple matrix, thereby improving the model’s generalization ability. Therefore, in practical applications of mixed pesticide detection, the NIR and SERS feature-level fusion method can maintain high detection accuracy even under the influence of the apple matrix, demonstrating stronger adaptability and greater potential for wider application.

## 5. Discussion

This study presents a food pesticide residue detection model based on Near-Infrared Spectroscopy (NIR) and Surface-Enhanced Raman Spectroscopy (SERS) technologies. By integrating the spectral information from both techniques, we achieved efficient and precise detection of pesticide residues in food. Compared to existing studies in this field, our approach and results show significant advantages. For example, Pham et al. (2022) developed a hydrophobic polysiloxane-modified silver nanoparticle substrate for SERS applications, which was used for detecting pesticide residues in mangoes, proposing a rapid screening method [39]. In contrast, our method not only shares similar advantages in rapid screening but more importantly, through the combination of NIR and SERS spectral information, significantly improves the detection accuracy and sensitivity, making pesticide residue detection more efficient and reliable.

The contributions of this study are primarily reflected in the following aspects: First, by combining NIR and SERS spectral techniques, a new spectral data fusion method is proposed, enabling complementary information from both techniques to enhance pesticide residue detection precision and sensitivity. Second, the introduction of the HSIC-VSIO algorithm for spectral feature selection and fusion overcomes the limitations of single-spectral technologies, improving the performance of the detection model. The multivariate calibration model developed in this study not only enriches the theoretical application of spectral analysis techniques but also provides a more reliable tool for practical detection. Moreover, it expands the application prospects of spectral analysis technology in food safety testing, advances the development of technology in this field, and offers new insights into the development of pesticide residue detection methods. This research holds significant implications for ensuring food safety and public health.

## 6. Conclusion

This study investigated a food pesticide residue detection method based on Near-Infrared Spectroscopy (NIR) and Surface-Enhanced Raman Spectroscopy (SERS) technologies. By integrating spectral information from both techniques and employing the Hilbert-Schmidt Independence Criterion-based Variable Space Iterative Optimization (HSIC-VSIO) algorithm for feature variable selection, we developed a highly efficient and sensitive multivariate calibration model. The research findings indicate the following:

(1) By integrating NIR and SERS spectral data and employing a feature-level fusion approach, the accuracy and sensitivity of pesticide residue detection were significantly improved. Experimental results showed that the feature-level fusion model achieved a root mean square error of calibration (RMSE1) of 0.160, a root mean square error of prediction (RMSE2) of 0.185, a prediction coefficient of determination (R²) of 0.988, and a relative prediction deviation (RPD) of 8.290.
(2) Compared to single spectral techniques, the NIR-SERS feature-level fusion method exhibited the lowest errors in both calibration and prediction sets, the highest determination coefficients, and a significantly higher RPD than other methods, fully demonstrating its superiority and application potential in detecting pesticide residues in complex matrices.
(3) The HSIC-VSIO algorithm introduced in this study effectively selected and fused spectral feature variables, overcoming the limitations of single spectral techniques and significantly enhancing the performance of the detection model, providing a more reliable tool for practical applications.
(4) SERS technology demonstrated high sensitivity in detecting low concentrations of chlorpyrifos and pyrimethanil. Even at a concentration of 10 ⁻ ⁶ M, key characteristic peaks remained detectable. The NIR-SERS combined analysis method not only improved the accuracy of pesticide residue detection but also effectively mitigated interference from food matrices, ensuring high model stability and accuracy in complex sample environments.

Despite the significant findings of this study, some limitations remain. First, the variety and number of experimental samples were relatively limited; future studies should expand the sample range to verify the generalizability of the method. Second, while the NIR-SERS spectral fusion method performed well under laboratory conditions, environmental factors may impact its practical application, necessitating further model optimization to enhance robustness. Finally, future research could explore the integration of additional spectral technologies or sensors to further improve detection performance. Expanding the experimental sample set to include different types of agricultural products would help validate the method’s effectiveness and stability in broader applications. Moreover, optimizing multivariate calibration models to address potential environmental interferences in practical detection scenarios would enhance robustness in complex conditions. Considering current technological advancements, incorporating machine learning and artificial intelligence could facilitate the development of intelligent detection systems for real-time, online pesticide residue monitoring.

## Supporting information

S1 Data Figure 3 Raman spectra.xlsx: Provides the source data for drawing this picture. (XLSX)

S2 Data Figure 4 Conventional Raman spectra of chlorpyrifos and pymetrozine solutions.xlsx: Provides the source data for drawing this picture. (XLSX)

S3 Data Figure 5 Conventional Raman spectra of OPP solution.xlsx: Provides the source data for drawing this picture. (XLSX)

S4 Data Figure 6 SERS spectra of standard solutions of chlorpyrifos, pymetrozine, and their equimolar mixture.xlsx: Provides the source data for drawing this picture. (XLSX)
